# Symptom duration and postoperative outcomes in degenerative cervical myelopathy: a systematic review and meta-analysis

**DOI:** 10.1007/s10143-026-04543-z

**Published:** 2026-10-17

**Authors:** Michał Szymoniuk, Wojciech Czyżewski, Marek Kochański, Barbara Buchalska, Piotr Kamieniak

**Affiliations:** 1 Doctoral School of the Medical University of Lublin, Medical University of Lublin, Lublin, Lubelskie Voivodeship Poland; 2 Department of Neurosurgery and Pediatric Neurosurgery, Medical University of Lublin, Lublin, Lubelskie Voivodeship Poland; 3 Department of Neurosurgery, T. Marciniak Lower Silesian Specialist Hospital - Emergency Medicine Center, Wrocław, Lower Silesian Voivodeship Poland; 4 Department of Neurosurgery, Maria Sklodowska-Curie National Research Institute of Oncology, Warsaw, Masovian Voivodeship Poland; 5 Department of Didactics and Medical Simulation, Medical University of Lublin, Lublin, Lubelskie Voivodeship Poland; 6 Faculty of Medicine, Medical University of Warsaw, Warsaw, Masovian Voivodeship Poland

**Keywords:** Degenerative cervical myelopathy, Symptom duration, Surgical timing, mJOA score, Neurological recovery, Meta-analysis

## Abstract

Background: Surgical decompression is regarded as standard treatment for degenerative cervical myelopathy (DCM). However, the timing of surgery remains a critical yet unresolved issue in clinical practice. Therefore, this meta-analysis aimed to evaluate the impact of symptom duration on surgical outcomes and determine the optimal timing for surgery. Methods: A systematic search was conducted on 16th November 2025 using PubMed, Ovid, Scopus, and Web of Science databases to identify eligible articles. Studies comprising patients with DCM undergoing surgery, assessing outcome by mJOA score at a minimum of 12 months of follow-up, were included. A risk of bias in the included studies was assessed using the Quality in Prognostic Studies (QUIPS) tool. Results: From 1522 records, 14 studies encompassing a total of 3162 patients were included in the current meta-analysis. Symptom duration was significantly associated with postoperative mJOA score according to univariate (OR: 2.15; 95% CI: 1.64–2.81) and multivariate data (OR: 1.78; 95% CI: 1.23–2.57). Meta-regression showed that neither baseline mJOA score nor age affected the association. Moreover, early surgery resulted in reduced neck pain and disability measured by the NDI score (OR: 1.56; 95% CI: 1.24–1.95), but not quality of life according to EQ-5D (OR: 1.47; 95% CI: 0.74–2.89). At the 6-month and 12-month cut-off points, patients with longer symptom duration had significantly worse outcomes in terms of mJOA score, and after exclusion of high-risk-of-bias studies, only at the 12-month cut-off point. However, the test for subgroup differences was insignificant (p = 0.89). Conclusion: Longer symptom duration is associated with poor surgical outcomes. Therefore, the duration of symptoms should be regarded as a clinically meaningful factor when counseling patients with DCM and planning surgical intervention. An optimal threshold for symptom duration, indicating when the surgery should be performed to prevent poor outcomes, cannot be recommended due to limitations of published data and needs to be established by further well-designed prospective studies. Future research should also aim to define optimal time cut-offs across different degrees of DCM severity and varied disease trajectories.

**Supplementary Information:**

The online version contains supplementary material available at https://doi.org/10.1007/s10143-026-04543-z.

## Introduction

 Degenerative cervical myelopathy (DCM) represents one of the most common causes of spinal cord dysfunction, characterized by a progressive course [47]. The initial symptoms are usually subtle, including numbness and loss of dexterity in the fingers [20, 50]. With the progression of the disease, symptoms such as gait disturbance and weakness in the upper extremities may appear [20, 50]. Finally, most severe forms of DCM are characterized by quadriparesis and sphincter incontinence [20, 50].

The surgical decompression constitutes the treatment of choice for patients with DCM [11]. Since the surgery can halt the progression of the disease, its importance is widely accepted by experts and clinicians, even if the functional status and quality of life do not improve after treatment in some patients [15]. On the other hand, several studies suggested that myelopathy severity improved better following surgery in patients with a shorter duration of symptoms than in patients whose disease lasted longer [13, 46, 51]. Considering the duration of symptoms in treatment planning could potentially improve its results, leading to a better quality of life and greater satisfaction in treated patients. However, current evidence does not clearly answer when the surgery should be performed in patients with DCM to obtain the best treatment outcomes [42].

To date, numerous cohort studies have investigated the association between symptom duration and surgical outcomes in DCM. However, some studies have found a significant correlation, while others have suggested otherwise. Moreover, the optimal timing of surgical intervention in DCM has not been established. Hence, the current meta-analysis aimed to (1) explore the association between symptom duration and outcomes following surgery for DCM, and (2) assess the optimal timeframe for surgical intervention, the exceeding of which results in significantly worse outcomes.

## Methods

This systematic review and meta-analysis were prepared according to the Preferred Reporting Items for Systematic Reviews and Meta-Analyses (PRISMA) statement (Supplementary File No. 1) [36]. The review protocol was registered in the International Prospective Register of Systematic Reviews (PROSPERO) under the registration number: CRD420251119564.

### Information sources and search strategy

A systematic search was conducted on 16th November 2025 using MEDLINE (via PubMed and Ovid platforms), Scopus, and Web of Science databases to identify eligible articles published between their inception and the date of search. The search strategy was developed by the authors for the advanced search engine of the PubMed platform and adapted to other platforms using the Polyglot Search Translator tool (Table 1) [21]. Additionally, the reference lists of articles included in the systematic review were hand-searched for potentially eligible studies, which the applied search strategy had omitted.

**Table 1:** PubMed search strategy

Set No.	Concept	Search query
1	Cervical	“Cervical Vertebrae“[Mesh] OR “Cervical Cord“[Mesh] OR cervical[ti]
2	Myelopathy	myelopath*[ti] OR myeloradiculopath*[ti] OR spondylomyelopath*[ti] OR spondylomyelopath*[ti] OR “Spinal Cord Compression“[Mesh] OR “degenerative cervical myelopathy“[ti] OR “cervical spondylotic myelopathy“[ti] OR “cervical compressive myelopathy“[ti]
3		1 AND 2
4	Exclude	“Atlanto-Occipital Joint“[Mesh] OR “Arteriovenous Fistula“[Mesh] OR “Radiotherapy“[Mesh] OR “Vitamin B 12“[Mesh] OR “Radiation“[Mesh] OR “Radiation Injuries“[Mesh] OR “Re-Irradiation“[Mesh] OR “Craniospinal Irradiation“[Mesh] OR “Motor Neuron Disease“[Mesh] OR “Amyotrophic Lateral Sclerosis“[Mesh] OR “Neoplasm Metastasis“[Mesh] OR “Hemangioma“[Mesh] OR metastasis[tiab] OR neoplasm[tiab] OR tumor*[tiab] OR “Nervous System Malformations“[Mesh] OR “Autoimmune Diseases of the Nervous System“[Mesh] OR “Congenital, Hereditary, and Neonatal Diseases and Abnormalities“[Mesh] OR “Virus Diseases“[Mesh] OR “Spinal Cord Injuries“[Mesh] OR “Trauma, Nervous System“[Mesh]
5		3 NOT 4
6	Surgical treatment	surg*[tiab] OR “Surgical Procedures, Operative“[Mesh] OR Diskectomy[Mesh] OR discectomy[tiab] OR “anterior cervical discectomy“[tiab] OR “anterior cervical discectomy and fusion“[tiab] OR “cervical laminoplasty“[tiab] OR laminoplasty[tiab] OR laminectomy[tiab] OR laminotomy[tiab] OR corpectomy[tiab] OR “anterior cervical corpectomy“[tiab] OR “anterior cervical corpectomy and fusion“[tiab] OR “360-degree“[tiab] OR “360 degree“[tiab]
7	Duration of symptoms	“symptom* duration“[tw] OR “symptom* length“[tw] OR “disease duration“[tw] OR “delay in diagnosis“[tw] OR “duration of symptom*“[tw] OR “duration of disease“[tw]
8	Predictors	predict*[ti] OR prognos*[ti] OR correlat*[ti] OR associat*[ti] OR influence*[ti] OR impact*[ti] OR “risk factor*“[ti]
9		7 OR 8
10	Neurological outcome	outcome*[tiab] OR recovery[tiab] OR “neurological improvement*“[tiab] OR mJOA[tiab] OR JOA[tiab] OR “functional improvement*“[tiab] OR “postoperative improvement“[tiab] OR “post*operative efficacy“[tiab] OR “post*operative improvement“[tiab]
11	Exclude	“quality of life“[ti] OR complication*[ti] OR “cost effectiveness“[ti] OR pain[ti] OR alignment[ti] OR deformity[ti] OR lordo*[ti] OR kypho*[ti] OR “patient* satisfaction“[ti] OR adverse[ti] OR mortality[ti] OR “patient-reported outcome“[ti] OR “signal intensity“[ti] OR occupational[ti] OR work[ti]
12		10 NOT 11
13		5 AND 6 AND 9 AND 12
14	Filters	english[Filter] or polish[Filter]
15	Filters	humans[Filter]
16		13 AND 14 AND 15

### Selection process

A complete dual review process was conducted, involving the screening of titles and abstracts, as well as full texts, independently by two authors (M.K., M.S.). Disagreements between the assessments were solved through discussion. No automation tools have been used during the screening process.

### Eligibility criteria

Eligible records had to meet the following inclusion criteria:

- randomized controlled trials (RCTs), prospective or retrospective cohort studies, case-control studies.
- adults with DCM undergoing surgical treatment.
- duration of symptoms as one of the studied predictors of surgical outcome, given as a categorical variable.
- neurological recovery as one of the outcomes, measured by mJOA score as a dichotomous variable.
- minimum 12 months of follow-up.
- written in English or Polish.

The records were excluded if reported:

- case series, case reports, cross-sectional studies, reviews, systematic reviews, and meta-analyses.
- editorials, communications, and letters to the editor.
- duration of symptoms as a continuous variable.
- insufficient data for quantitative analysis.
- less than 12 months of follow-up.
- preclinical studies.
- unpublished studies (e.g., conference abstracts, trial protocols, preprints).
- studies with overlapping populations.

### Data extraction

Data from eligible studies were extracted by two authors independently (M.S., W.C.) according to predefined domains: study design, location of study, number of participants, mean age, mean follow-up duration, surgical technique(s) used, cut-off point(s) between short and long symptom duration, type of predictor analysis used (univariate or multivariate), reported outcomes, and the results of correlation analysis between symptom duration and surgical outcomes. The primary outcome was postoperative neurological function(measured by mJOA score). The secondary outcomes included postoperative disability (measured by Neck Disability Index (NDI) questionnaire) and postoperative quality of life (measured by EuroQol 5 Dimensions (EQ-5D) questionnaire). Odds ratios (ORs) for the associations between symptom duration and the outcomes from the last available follow-up were extracted. Any disagreements between the authors were resolved through discussion.

If several articles reported the results of the same study or were based on the same study population, the article reporting the best-fitting data for quantitative analysis was chosen, and other articles were excluded. If that criterion was met in more than one article, the last published report was included.

### Risk of bias assessment

The Quality in Prognostic Studies (QUIPS) instrument was used to assess risk of bias within eligible studies [14]. Articles were assessed as having a high, unclear, or low overall risk of bias across six domains: (1) study participation, (2) study attrition, (3) prognostic factor measurement, (4) outcome measurement, (5) study confounding, and (6) statistical analysis and reporting. If the study had a high risk of bias in at least one domain, the overall risk of bias was reported as high. Studies with low risk of bias in all domains were considered to have low overall risk of bias. In other cases, the overall risk of bias was rated as unclear. The risk of bias assessment was conducted independently by two authors (B.B., M.K.). Disagreements between the assessments were resolved by discussion with the other author (M.S.). If the article missed data important for risk of bias assessment, the study investigators were contacted to obtain the necessary information.

### Effect measures

Analysis of each dichotomous outcome was carried out by calculating the OR of a poor outcome defined by the included studies. Poor outcome of surgery was defined as a fair mJOA recovery rate, or not achieving MCID of mJOA score, depending on how the authors reported the outcomes. For NDI and EQ-5D, the same definition of poor outcome has been applied. If the study provided an OR of a successful outcome, it was recalculated to an OR of a poor outcome. Subgroup analysis of different cut-offs of symptom duration was performed to test whether optimal surgical timing can be determined. A meta-regression was conducted to examine the influence of disease severity, age, and other factors on the association between symptom duration and surgical outcome.

### Data synthesis and statistical analysis

Quantitative analysis was performed for outcomes with a minimum of two eligible studies. Otherwise, the data were analyzed qualitatively and presented in narrative form. For quantitative synthesis, adjusted ORs derived from multivariate regression models were used when available. If no adjusted OR was reported, unadjusted (univariate) ORs were used. If the ORs were not reported in eligible articles, the ORs were calculated from the available data (number of patients in each group, dichotomized cut-offs). In the remaining cases, the authors of each study were contacted to obtain the missing data. If possible, a separate meta-analysis of ORs from univariate and multivariate analyses was planned. Otherwise, when the number of studies precluded performing separate analyses, the ORs from univariate and multivariate analyses were pooled in one meta-analysis. The results of each meta-analysis were presented in the form of forest plots.

Statistical analysis was performed using R 4.5.1 (R Core Team) [39], the *meta* (*v.8.2-0*; Balduzzi, Rücker & Schwarzer) [5], the *robvis* (*v.0.3.0*; McGuinness and Higgins) [29], and the *tidyverse* (*v.2.0.0*; Wickham) [49] packages. Estimates of ORs with a 95% confidence interval were calculated for the overall population of all included patients, as well as for subgroups based on the duration of symptoms. The inverse-variance method was used in all meta-analyses. Due to expected substantial or high clinical and methodological heterogeneity among eligible studies (different cut-off points of symptom duration, different surgical techniques), the random effects model was used in all analyses to pool effect estimates, allowing for variation in the true effects across studies. The Paule and Mandel estimator [37] was used to estimate the between-study variance due to its favourable statistical properties in binary effect size data [4].

Heterogeneity was assessed by the I² statistic and Cochran’s Q test. Substantial heterogeneity was detected when I^2^ was above 60%, as well as when the p-value of the Q-test was below 0.05. In case of detected substantial heterogeneity, subgroup analyses or meta-regression were conducted to search for its source for analyses encompassing more than ten studies. In case of high heterogeneity (I^2^ > 75%), the meta-analysis was not conducted, and the data were analyzed in narrative form.

To verify the robustness of the obtained results and check whether the results were influenced by a single study, a leave-one-out sensitivity analysis was conducted for each analysis. Moreover, studies at high risk of bias were excluded to check if study quality influenced the obtained results,

### Reporting bias assessment

Publication bias was assessed by visual inspection of contour-enhanced funnel plots [38] and Egger’s regression test [10]. Assessment was conducted only if the number of studies included for a particular meta-analysis exceeded 10. Statistically significant publication bias was considered when the p-value < 0.1.

## Results

### Study selection

**Fig. 1:**
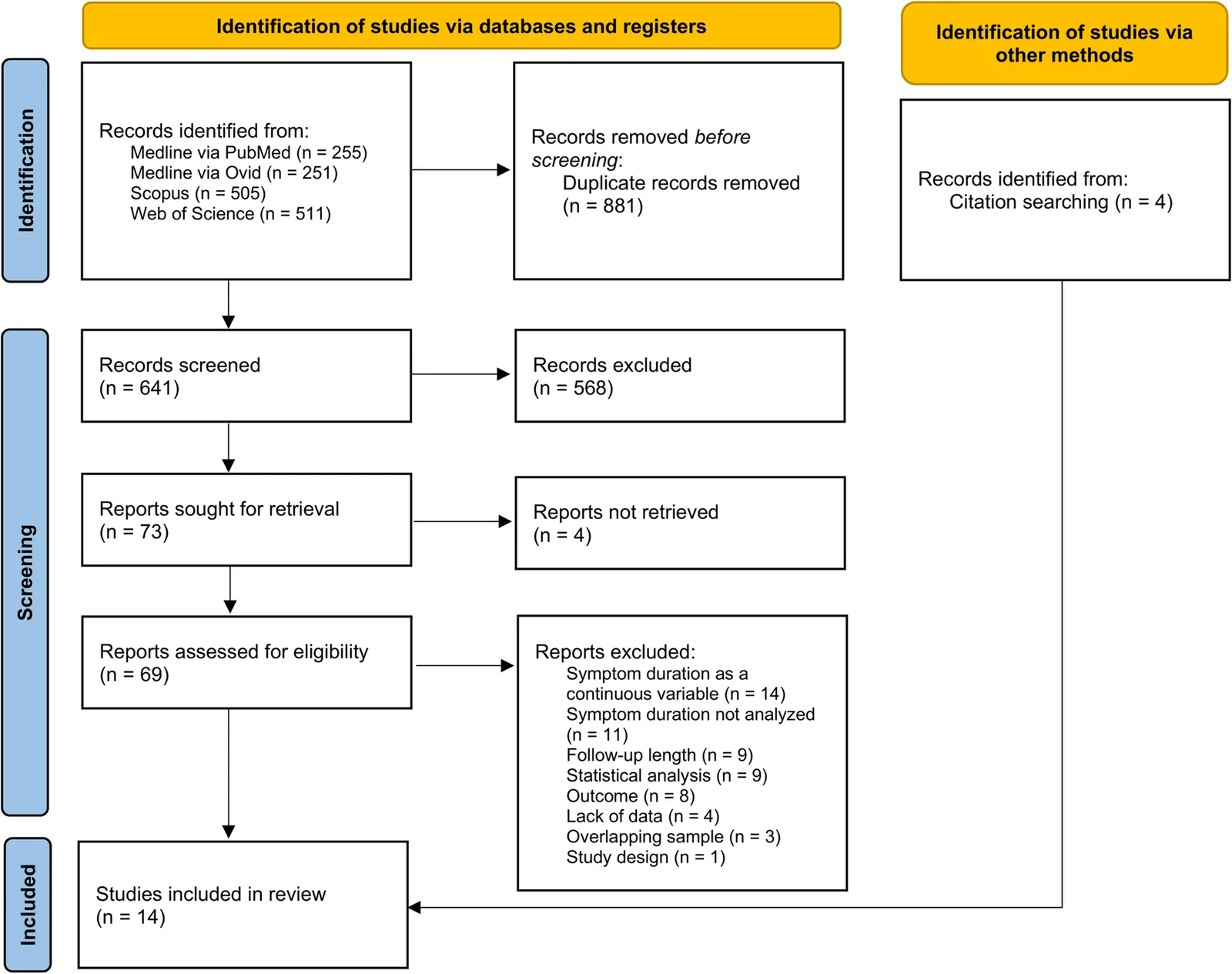
PRISMA 2020 flow diagram presenting the results of the study search and selection process

The initial search identified a total of 1522 records, including 255 from PubMed, 251 from Ovid, 505 from Scopus, and 511 from Web of Science. After removing 881 duplicates, 641 records were screened by titles and abstracts. The high duplication rate (58%) reflects substantial overlap between PubMed and Ovid records, as both platforms provide access to the same MEDLINE database. Of 73 eligible records, full texts of four were not retrieved. According to the results of full-text screening, 59 of 69 screened records were excluded due to the following reasons: symptom duration was treated as a continuous variable (14), symptom duration was not analyzed as a predictor of surgical outcome (11), follow-up duration was shorter than one year (9), statistical analyses used in the study were not suitable for planned meta-analyses (9), the study outcome was inappropriate (8), lack of data needed for quantitative analysis (4), there was serious concern that study population overlapped with other eligible studies (3), study design was not appropriate (1). Four additional articles were identified from citation hand-searching of reference lists. Finally, 14 studies met eligibility criteria and were included in the current systematic review [1, 2, 6, 7, 9, 12, 24–27, 30, 31, 35, 43] (Fig. 1).

### Study characteristics

Fourteen studies (3162 patients) were included in the current systematic review (Table 2). Nine studies were retrospective [1, 2, 7, 12, 25, 26, 30, 31, 35], two studies were retrospective from prospectively collected data [6, 9], and the remaining three were prospective [24, 27, 43]. The mean age of patients in qualified studies ranged from 51 ± 7.3 years to 66.6 ± 10.1 years. Outcome was assessed by mJOA recovery rates (calculated using the Hirabayashi method [16]) in eight studies [7, 12, 26, 27, 30, 31, 35, 43], raw mJOA scores in four studies [2, 6, 9], and the minimal clinically important difference (MCID) for mJOA in three studies [1, 24, 25]. Besides mJOA, three studies evaluated NDI [6, 24, 25], one study evaluated the Nurick scale [30], and two studies adopted the EQ-5D questionnaire [6, 24] as an additional outcome measure. Eligible studies were conducted in China (3) [7, 12, 26], United States (2) [6, 25], India (2) [1, 2], Japan (2) [27, 35], Canada (1) [24], Iran (1) [31], Italy (1) [43], Turkey (1) [30], and one study was conducted worldwide [9].

**Table 2:** Characteristics of included studies

Author	Year	Country	Study design	No. of patients	Mean age (years)	Cut-off point(s) between short and long duration of symptoms (months)	Surgical approach	Mean follow-up (months)	Study outcomes	Reported definition of outcome	Type of factor analysis	Confounding factors in multivariate analysis
Levett	2025	Canada	prospective cohort	483	60,14 ± 11,2	3, 6, 12, 24	ACDF, ACCF, laminoplasty, laminectomy, laminectomy with fusion, 360-degree approach	12	mJOA MCID, NDI, EQ-5D, SF-12, NRS for neck, NRS for arm	good outcome; achieving MCID (> 3 for severe, > 2 for moderate,> 1 for mild)	multivariate	BMI, number of comorbidities, smoking, mJOA, NRS for neck pain
Chan	2024	United States	retrospective cohort (from prospectively collected data)	948	NA	3, 12	NA	24	mJOA score, NDI, EQ-5D	good outcome; achieving top 20th percentile of score change	multivariate	BMI, type of insurance, smoking, comorbidities (CAD, anxiety, arthritis, COPD), presence of myelopathy, pain location, presence of motor deficit, ability to ambulate, workers’ compensation, employment status, laminoplasty, ACDF, VAS neck pain, VAS radicular arm pain, NDI, EQ-VAS, EQ-5D, mJOA
Meral	2024	Turkey	retrospective cohort	32	66.3 ± 8.5	6, 18	laminoplasty	34.7	mJOA recovery rate, Nurick scale	good outcome; complete recovery according to Hirabayashi’s recovery rate	univariate	-
Acharya	2023	India	retrospective cohort	124	61.5 ± 13.2	6	ACDF, ACCF, laminectomy, laminectomy with fusion, laminoplasty, 360-degree approach	18.7	mJOA MCID	poor outcome; missing MCID (< 3 points)	multivariate	preoperative mJOA, MRI signal changes, change in cSVA
Levy	2023	United States	retrospective cohort	98	59.2 ± 10.9	6, 24	laminectomy with fusion	17.1	mJOA MCID, VAS for neck, VAS for arm, NDI, SF-12	good outcome; achieving MCID (> 2.71 points)	multivariate	sex, smoking
Moradi	2023	Iran	retrospective cohort	157	63.4 ± 6.2	12	laminectomy with fusion	25	mJOA recovery rate	poor outcome; recovery rate < 75%	multivariate	age, MRI signal changes, diabetes, mJOA
Li	2022	China	retrospective cohort	137	57.6 ± 12.2	12	anterior, posterior, 360-degree approach	42.6	mJOA recovery rate	poor outcome; recovery rate < 50%	multivariate	sex, age, smoking, mJOA, CAD
Machino	2021	Japan	prospective cohort	505	66.6 ± 10.1	6	laminoplasty	26.5	mJOA recovery rate	poor outcome; recovery rate < 50%	univariate	-
Severino	2020	Italy	prospective cohort	36	57.1 ± 13.2	6	ACDF, ACCF, laminectomy, laminectomy with fusion	12	mJOA recovery rate	good outcome; recovery rate > 50%	univariate	-
De La Garza	2019	Worldwide	retrospective cohort (from prospectively collected data)	251	57.5 ± 12	4, 12	ACDF, ACCF, laminectomy, laminectomy with fusion, laminoplasty	24	mJOA score	good outcome; return to normal neurologic function (mJOA score of 18)	multivariate	age, psychiatric comorbidities, gait impairment, OPLL, OLF, compression source, MRI signal changes (T2 and T1), severe myelopathy, mJOA, 30 m walking test
Aggarwal	2016	India	retrospective cohort	78	NA	12	ACDF, ACCF, laminectomy	NA	mJOA score	good (mJOA score ≥ 16) and poor outcome (mJOA score < 16)	univariate	-
Oichi	2016	Japan	retrospective cohort	125	64.2 ± 11	12	laminectomy	24	mJOA recovery rate	poor outcome; recovery rate < 50%	multivariate	age, sex, diabetes, listhesis
Chung	2012	China	retrospective cohort	43	65.3 ± 8.5	12	laminoplasty	84	mJOA recovery rate	good outcome; recovery rate ≥ 50%	univariate	-
Gao	2012	China	retrospective cohort	145	51 ± 7.3	12	ACCF	102.1	mJOA recovery rate	poor outcome; recovery rate ≤ 50%	multivariate	MRI signal changes, mJOA

*ACCF* anterior cervical corpectomy and fusion, *ACDF* anterior cervical discectomy and fusion, *BMI* body mass index, *CAD* carotid artery disease, *COPD *chronic obstructive pulmonary disease, *cSVA* cervical sagittal vertical axis, *MCID* minimal clinically important difference, *mJOA* modified Japanese Orthopaedic Association scale, *NDI* Neck Disability Index, *NRS* Numeric Rating Scale, *OLF* ossification of ligamentum flavum, *OPLL* ossification of posterior longitudinal ligament, *VAS* visual analog scale, *NA* not reported

### Risk of bias assessment

**Fig. 2:**
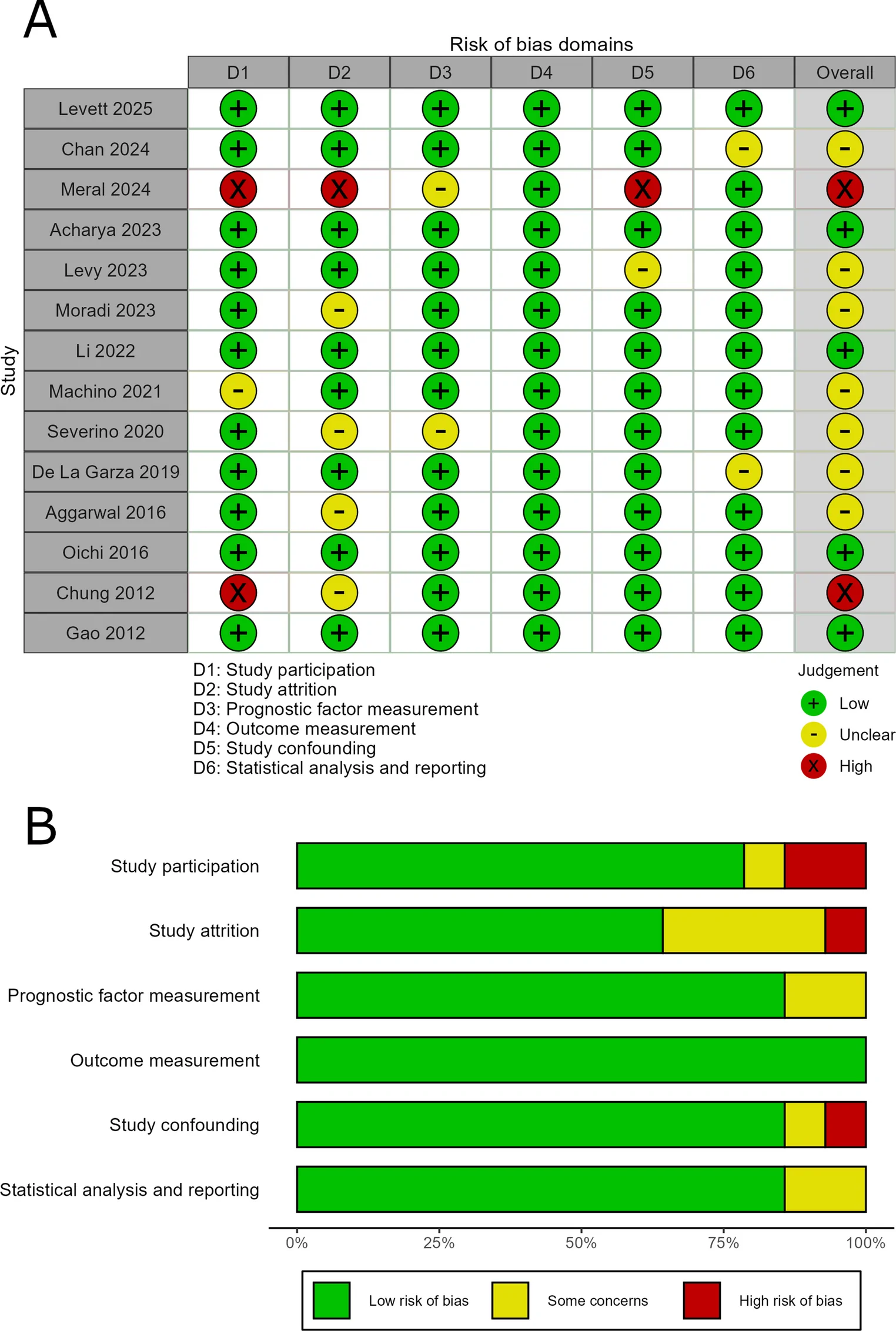
Results of the risk of bias assessment according to the QUIPS tool: (**A**) traffic-light plot; (**B**) risk of bias summary plot

The results of the risk of bias assessment according to the QUIPS tool were presented in Fig. 2 (Fig. 2). In terms of risk of bias, some concerns were present in nine [2, 6, 7, 9, 25, 27, 30, 31, 43] of 14 studies, including two studies with an overall high risk of bias [7, 30].

### Impact of symptom duration on surgical outcomes

#### Primary outcome

**Fig. 3:**
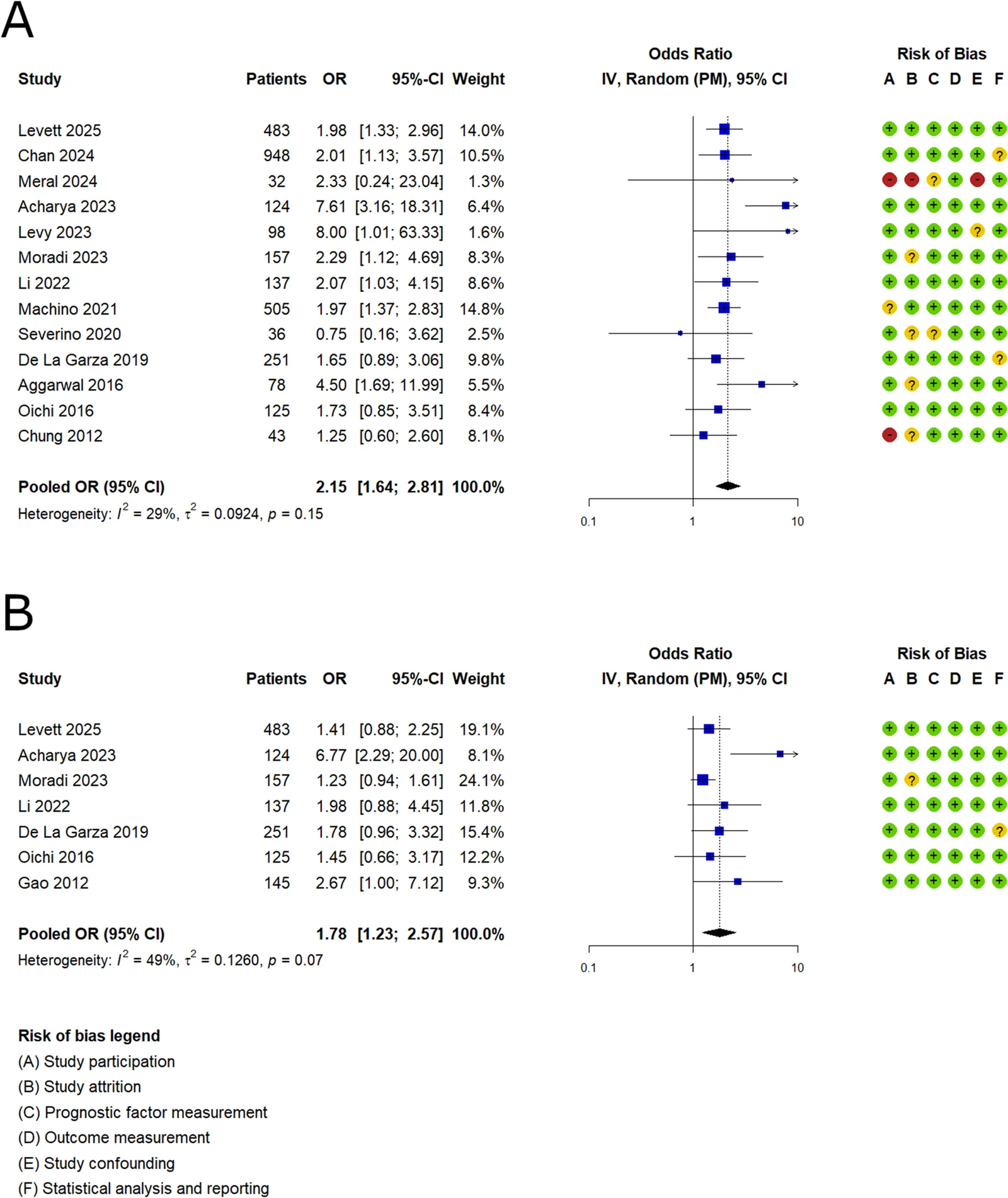
Forest plots showing pooled odds ratios of association between symptom duration and poor surgical outcome measured by mJOA score: (**A**) meta-analysis of univariate data; (**B**) meta-analysis of multivariate data. Abbreviations: OR – odds ratio, CI – confidence interval

**Fig. 4:**
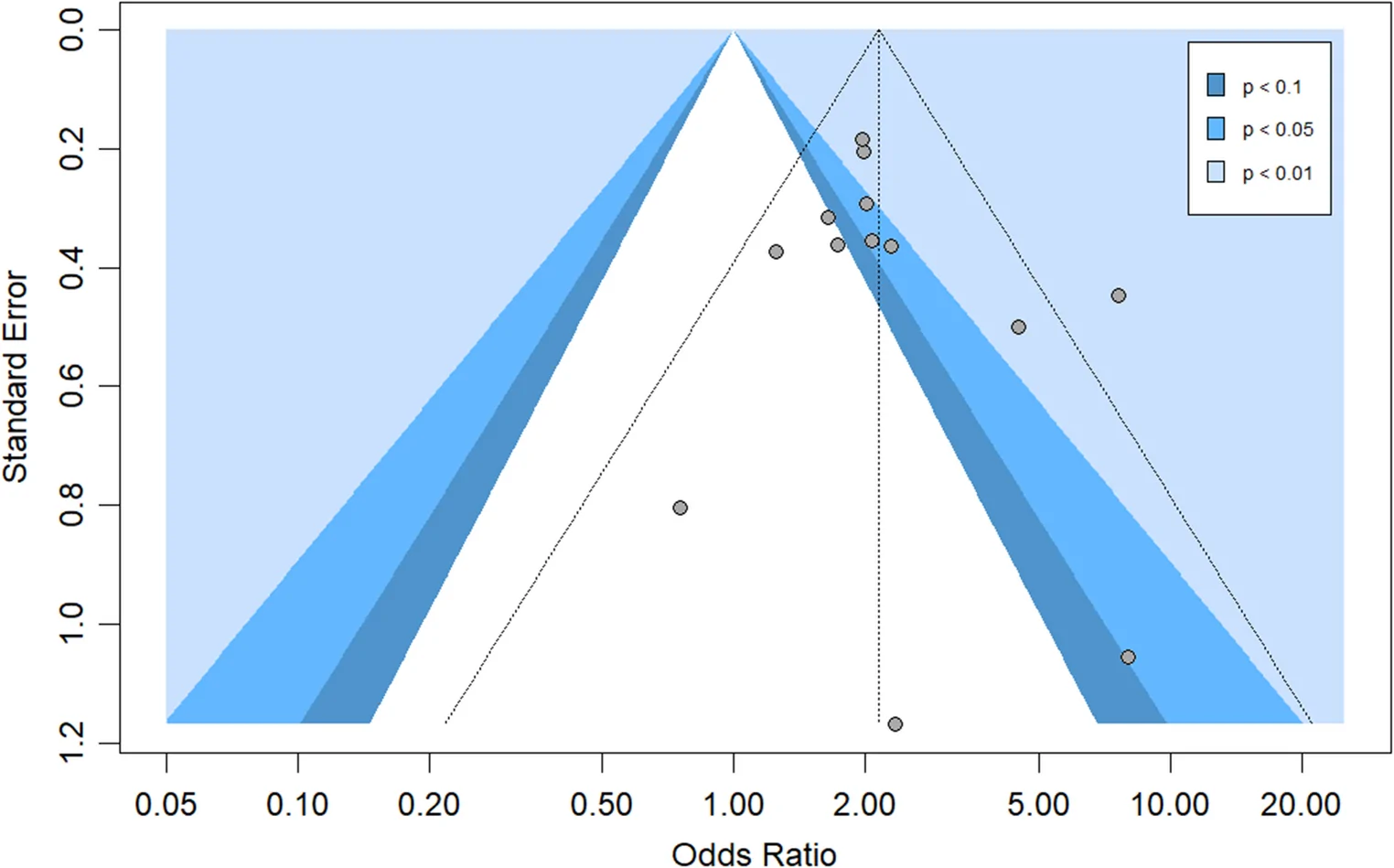
The funnel plot demonstrating the absence of significant publication bias among studies for univariate meta-analysis with the mJOA score as outcome

Univariate analysis of the association between symptom duration and postoperative mJOA score was performed by 13 studies [1, 2, 6, 7, 9, 24–27, 30, 31, 35, 43] (Fig. 3A). Four studies had a low risk of bias, seven had an unclear risk of bias, and two had a high risk of bias. No substantial heterogeneity was detected among these studies (I^2^ = 29%; *p* = 0.15). A longer symptom duration resulted in worse neurological outcome measured by the mJOA score according to the univariate data (OR: 2.15; 95% CI: 1.64–2.81). The visual inspection of the funnel plot demonstrated nearly symmetrical distribution of points on both sides of the vertical line, indicating no significant publication bias among studies (Fig. 4). The result of Egger’s test for funnel plot asymmetry confirmed that observation (t = 0.92, *p* = 0.37).

Seven studies [1, 9, 12, 24, 26, 31, 35] conducted multivariate analysis to evaluate the correlation of symptom duration and postoperative mJOA score (Fig. 3B). Five studies had an overall low risk of bias, and two studies had an unclear risk of bias. According to multivariate analyses, a longer symptom duration was significantly associated with a poor postoperative mJOA score (OR: 1.78; 95% CI: 1.23–2.57). The Cochran’s Q test and I^2^ statistic did not detect substantial heterogeneity (*p* = 0.07, I^2^ = 49%). Nevertheless, a meta-regression was performed to explore whether the association was modified by baseline severity or age (Table 3). Baseline severity did not significantly modify the association between symptom duration and surgical outcome (β = -0.119; 95%CI: -0.540-0.303; *p* = 0.581). Similarly, age did not influence the correlation between symptom duration and outcome (β = -0.043; 95%CI: -0.143-0.058; *p* = 0.402). Other assessed moderators, such as follow-up length and year of publication, did not reach statistical significance.

**Table 3:** Results of meta-regression exploring the impact of baseline severity and age on the association between symptom duration and postoperative mJOA score

Moderators	β coefficient	SE	*p* value	95% CI
Age	-0.043	0.051	0.402	-0.143 to 0.058
Baseline severity (mJOA)	-0.119	0.215	0.581	-0.540 to 0.303
Mean follow-up length	0.005	0.008	0.543	-0.011 to 0.021
Year of publication	-0.019	0.052	0.712	-0.121 to 0.082

Abbreviations: *mJOA* modified Japanese Orthopedic Association score

#### Secondary outcomes

**Fig. 5:**
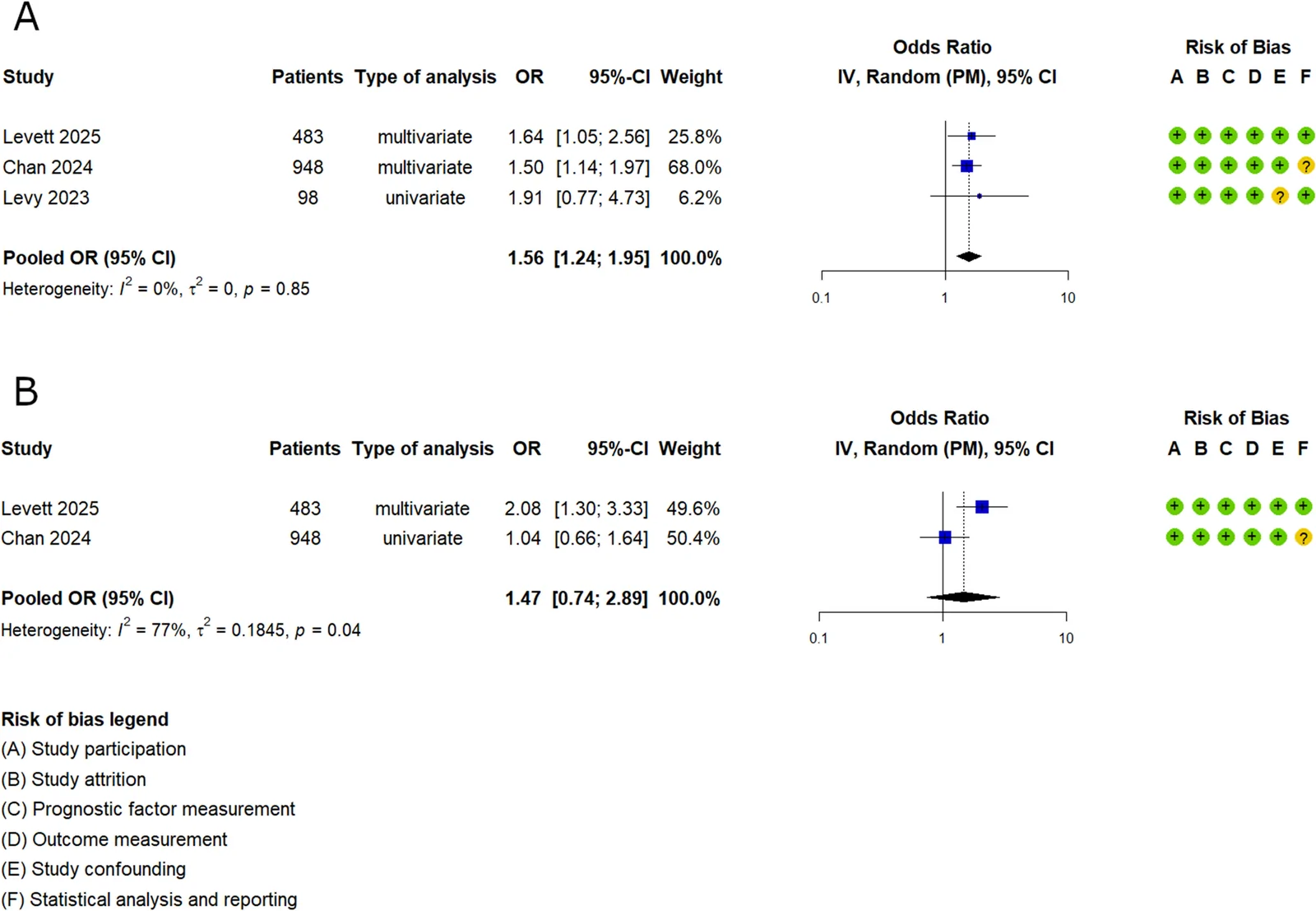
Forest plots showing pooled odds ratios of association between symptom duration and secondary outcomes: (**A**) neck disability index; (**B**) EuroQol 5 dimensions. Abbreviations: OR – odds ratio, CI – confidence interval

Three studies [6, 24, 25] evaluated the association between symptom duration and postoperative disability measured by NDI (Fig. 5A). One study demonstrated a low overall risk of bias, and two studies showed an unclear risk of bias. No substantial heterogeneity was detected among these studies (*p* = 0.85; I^2^ = 0.0%). Meta-analysis demonstrated that longer symptom duration was significantly correlated with a poor postoperative NDI score (OR: 1.56; 95% CI: 1.24–1.95).

Two studies investigated the influence of symptom duration on postoperative quality of life assessed by the EQ-5D questionnaire [6, 24] (Fig. 5B). At the end of the 24-month follow-up, the change in the EQ-5D score after surgical treatment did not differ among patients with symptom duration longer and shorter than 12 months (OR: 1.47; 95% CI: 0.74–2.89).

### Optimal timing of surgery in DCM

**Fig. 6:**
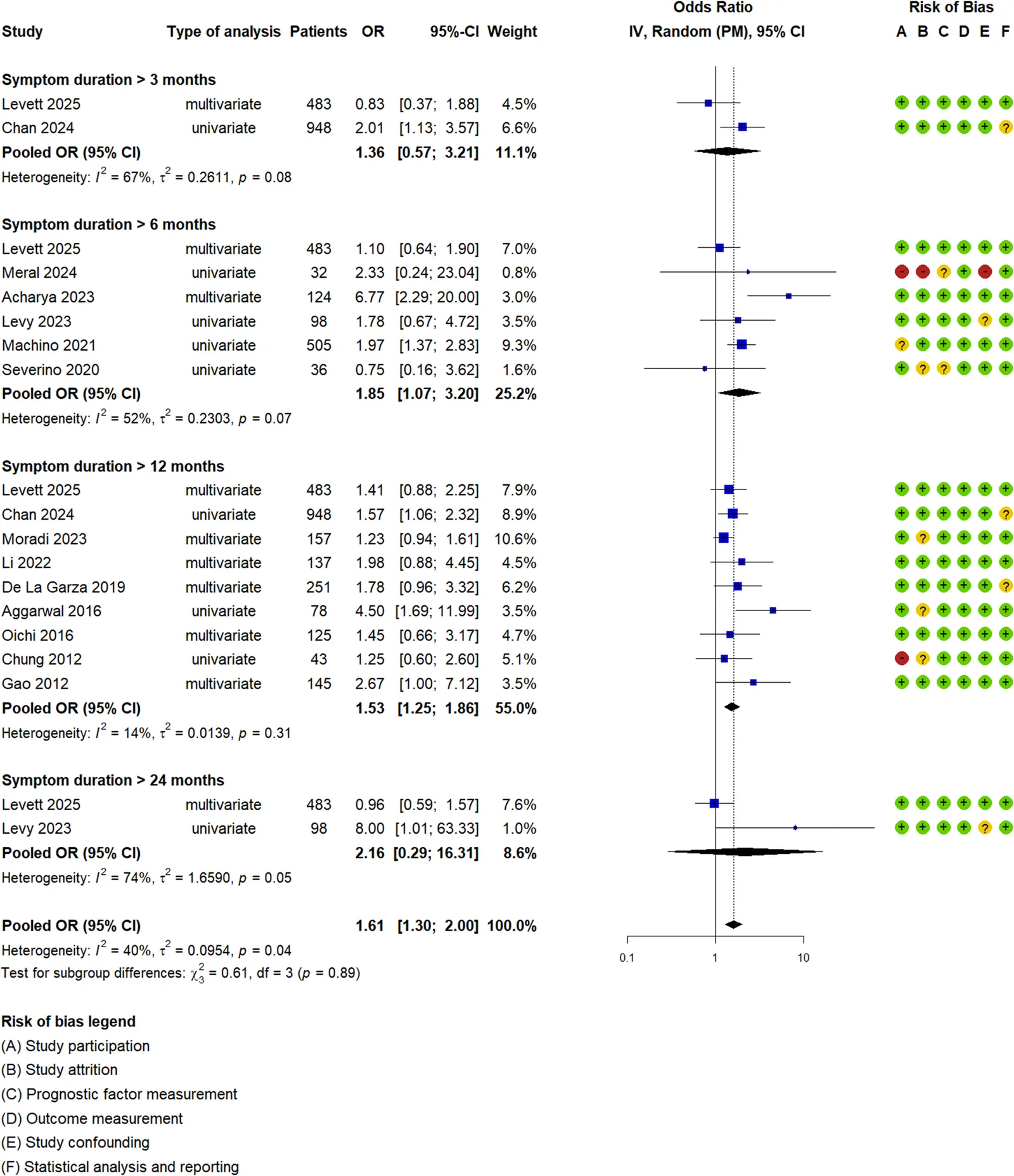
Forest plot showing the results of subgroup analysis on different cut-off points for short and long duration of symptoms in terms of neurological outcomes measured by the mJOA score. *Abbreviations*: OR – odds ratio, CI – confidence interval

Four cut-off points between short and long durations of symptoms were determined by the eligible studies, including 3 months (two studies [6, 24]), 6 months (six studies [1, 24, 25, 27, 30, 43]), 12 months (nine studies [2, 6, 7, 9, 12, 24, 26, 31, 35]), and 24 months (two studies [24, 25]) (Fig. 6). At the 12-month cut-off, patients with longer symptom duration had significantly worse outcomes in terms of mJOA score (OR: 1.53; 95% CI: 1.25–1.86; I^2^ = 14%). The difference between longer and shorter durations of symptoms regarding the change in mJOA score point was also statistically significant at the 6-month cut-off (OR: 1.85; 95% CI: 1.07–3.20; I^2^ = 52%). There was no difference in the improvement of neurological function assessed by the mJOA score between shorter and longer disease duration when surgery was performed within the first 3 months and 24 months of symptom duration. The test for subgroup differences was insignificant (*p* = 0.89), which suggests a lack of a specific cut-off point in symptom duration, exceeding which leads to poor surgical outcomes.

### Leave-one-out sensitivity analysis and results after exclusion of studies at high risk of bias

The leave-one-out analysis demonstrated that no single study had a large impact on the pooled OR and heterogeneity in almost all pooled analyses, confirming the robustness of the obtained results. However, in a meta-analysis of the mJOA score on multivariate data, omitting the study by Moradi et al. [31] resulted in a significant increase in the pooled OR (OR: 1.83; 95% CI: 1.37–2.43 vs. OR: 1.48; 95% CI: 1.22–1.80). Nevertheless, this study did not affect the significance between symptom duration and postoperative mJOA outcome. After excluding the studies at high risk of bias, the influence of symptom duration on the mJOA outcome in univariate analysis slightly increased (OR: 2.26; 95% CI: 1.68–3.05). In subgroup analysis, the difference between longer and shorter durations of symptoms regarding the change in mJOA score became insignificant at the 6-month cut-off point after exclusion of studies with high risk of bias (OR: 1.84; 95% CI: 0.96–3.53). The remaining analyses did not include high-risk-of-bias studies (Supplementary Material).

## Discussion

### Symptom duration and surgical outcome

In this systematic review and meta-analysis of 13 studies, a significant association between symptom duration and surgical outcome measured by the mJOA score was demonstrated in the analysis of univariate (OR: 2.15; 95% CI: 1.64–2.81) and multivariate data (OR: 1.78; 95% CI: 1.23–2.57) as well. Moreover, meta-regression showed that the association was not influenced by baseline severity of myelopathy or age. However, due to the limited number of studies, the results of the meta-regression should be interpreted as inconclusive, rather than confirmatory. Furthermore, our results demonstrated that early surgery is associated with reduced neck pain and disability measured by the NDI score (OR: 1.56; 95% CI: 1.24–1.95), but not quality of life according to EQ-5D (OR: 1.47; 95% CI: 0.74–2.89). However, due to the limited number of studies in pooled analyses with secondary outcomes, the quality of life and disability aspects need to be evaluated by future systematic reviews. Previously, a systematic review by Tetreault et al. demonstrated a significant influence of symptom duration on surgical outcomes [45]. However, in contrast to our review, the authors analyzed this association only qualitatively. Moreover, the knowledge gaps regarding optimal time threshold and the influence of disease severity, as indicated by the authors, were investigated by the current meta-analysis.

Available pathophysiological research may partially elucidate why patients undergoing late surgery experience worse outcomes than patients treated earlier. According to the existing hypotheses, mechanical compression directly leads to neuronal loss, axonal pathway disruption, and demyelination [19]. Additionally, through the increasing intramedullary pressure, the required blood supply of the spinal cord is impaired, resulting in ischemia, hypoxia, and further neuronal damage [19]. With the duration of the DCM, the pathobiological changes of the spinal cord may spread to segments adjacent to the compression site, which is known as ‘rostral and caudal degeneration’ [34]. Moreover, chronic compression results in architectural changes of the spinal cord microvasculature and abnormal distribution of the blood flow [22]. Furthermore, the proliferation of astrocytes and fibroblasts leads to scar tissue formation, which hinders axonal regeneration [19]. In addition, according to a study on a rat model of DCM, delayed surgical intervention may increase the risk of ischemia-reperfusion injury [48], a phenomenon associated with the overactivation of the immune system, resulting in neuronal oxidative damage [52].

### Optimal timing of surgery for DCM

The current evidence does not support a definitive threshold for optimal surgical timing in DCM. Among the evaluated thresholds, the 12-month cut-off (OR: 1.53; 95% CI: 1.25–1.86) and 6-month cut-off point (OR: 1.85; 95% CI: 1.07–3.20) demonstrated significant values. Moreover, after exclusion of studies at high risk of bias, only the 12-month cut-off point remained significant. However, since differences between various cut-off points in subgroup analysis were not statistically significant (*p* = 0.89), no cut-off can be statistically distinguished from the others. Therefore, the findings of the subgroup analysis should be regarded as exploratory rather than conclusive.

Compared to studies on lumbar degenerative disease, surgical timing for cervical spine conditions has been less studied [8, 17, 32, 41, 44]. A recent meta-analysis exploring the impact of symptom duration on outcomes of anterior cervical discectomy and fusion (ACDF) showed a significant association between symptom duration longer than 24 months and less improvement in NDI score (8.70 vs. 18.93 for shorter duration; *p* = 0.03), VAS for neck (1.95 vs. 3.33 for shorter duration; *p* = 0.003), and VAS for arm (1.74 vs. 3.72 for shorter duration; *p* < 0.001) [33]. In contrast, at 12-month and 6-month cut-off points, the results were not statistically significant, leading to the conclusion that the patients should undergo surgery within 2 years of symptom onset. However, the meta-analysis predominantly encompassed studies including subjects with cervical radiculopathy, with four studies assessing mixed populations with myelopathic patients. Most of the available studies, ineligible for the current systematic review and meta-analysis, demonstrated a significant association between symptom duration and DCM outcomes and suggested 4 months (3.3 points of mJOA change for short duration vs. 2.5 points; *p* = 0.0049) [46], 6.5 months (OR: 1.196; 95% CI: 1.135–1.261) [13], 23 months (AUC: 0.616; sensitivity: 67.4%; specificity: 53.5%) [28], and 32 months (2.8 points of mJOA change for short duration vs. 2.2 points; *p* = 0.029) [46] as optimal cut-offs. However, a large multicenter study encompassing 1036 patients from fourteen surgical centers found that symptom duration did not affect the postoperative mJOA and NDI scores [3]. Considering the findings from the previous studies and current meta-analysis, the evidence for an optimal cut-off for surgery in DCM remains conflicting. Although the natural history of DCM is characterized by slow, progressive neurological decline, the dynamics of deterioration may vary between patients [20]. Some individuals may also present with severe DCM despite only a short time having passed since disease onset [53]. These observations indicate that DCM patients constitute a heterogeneous population, whose optimal timing of surgery should be analyzed according to the pattern of disease course. Therefore, determining optimal timing thresholds considering different levels of myelopathy severity, which is another significant predictor of surgical outcomes [45], may solve the inconsistencies present in the available literature. Unfortunately, the lack of stratification of the population in included studies based on DCM severity precluded us from exploring the optimal timing for mild, moderate, and severe myelopathy.

### Clinical implications

The presented findings have direct implications for clinical decision-making in DCM. Symptom duration should be regarded as a clinically meaningful factor when counseling patients and planning surgical intervention. In practical terms, prolonged observation or delayed referral in patients with established myelopathic signs carries a measurable risk of limiting postoperative recovery, consistent with previous large-scale observational data, systematic syntheses, and current meta-analysis [45]. While individual disease trajectories differ and preclude uniform timing rules, the data support a low tolerance for avoidable delays once the diagnosis of DCM has been established. From the perspective of patient communication, the findings indicate that surgery should be discussed as a time-dependent intervention, even in the absence of overtly rapid neurological progression.

### Limitations

The current review demonstrated that available literature on the impact of symptom duration on surgical outcome in DCM is characterized by substantial heterogeneity and lacks well-designed studies. Most of them were retrospective and defined only one cut-off point in preoperative symptom duration or assumed the symptom duration as a continuous variable, aside from a few exceptions, such as the study by Tetreault or the study by Levett [24, 46]. In addition, most of the published studies did not clearly define what symptoms were considered as the first. Therefore, it is not known if some authors recorded the duration of disease from the onset of any spinal symptoms (e.g., neck or arm pain, symptoms characteristic of radiculopathy rather than DCM), or true ‘myelopathic’ symptoms such as gait disturbances. Moreover, measuring the duration of symptoms is prone to recall bias, since it is based on a patient’s report of when the first symptom occurred. Furthermore, the definition of poor outcomes varies across published studies, and almost every study used different dichotomization between poor and good outcomes, which significantly influenced between-study heterogeneity. Other limitations include various surgical approaches used in the same study and the presence of heterogeneous sets of confounders in multivariate analyses.

Consequently, limitations of the current meta-analysis stem from the above-described constraints of studies published to date. All included studies were non-randomized, cohort studies, and most of them were retrospective. Although the methodological quality of observational research is considered inferior in relation to RCTs, a great proportion of low risk of bias was observed in all domains of the QUIPS tool, ensuring reliability of the conducted analyses. However, the presence of a high risk of bias in study attrition, participation, and confounding in some of the included studies may have influenced the obtained results. Moreover, due to the limited number of cut-off points in symptom duration explored by included studies, it was impossible to obtain reliable subgroup analysis and determine a definitive surgical timing threshold. Additionally, despite attempts to contact the authors of included studies, data in some studies were missed due to selective reporting. In addition, only seven studies reported adjusted effect sizes in the meta-analysis of multivariate data, limiting the power of meta-regression. As a result, the absence of a statistically significant moderation effect should be interpreted cautiously. Moreover, the decision to include only studies reporting categorical symptom duration to enable threshold-based synthesis may have introduced reporting-style selection bias, and some part of the existing literature was omitted in the current review. Furthermore, the considerable variation in follow-up duration across studies (between 12 and 102 months) may have contributed to effect-size heterogeneity, as longer follow-up periods are more likely to capture late differences in the natural history of the condition. Finally, the studies published in languages other than English were excluded from analyses.

### Future directions

There is still insufficient empirical evidence to establish precise timing criteria based on the severity of the disease. Moreover, since the lack of statistically significant difference between different cut-offs in symptom duration could be caused by underpowering due to the limited number of studies, future research should explore cut-off points other than 12- and 6-month as in the study by Tetreault [46]. Furthermore, the natural history of disease may significantly impact the association between duration of symptoms and outcome. In the case of patients experiencing a rapid deterioration with a previous progressive or stable disease, establishing the optimal cut-off for surgery may play a more important role. Therefore, future studies may explore optimal timing for DCM severity groups (mild, moderate, severe), and different patterns of disease course (slowly and rapidly progressive), separately. Since none of the studies included reported the time between the onset of rapid progression and surgery, this limitation may also be addressed by future cohort studies. Further studies should also clearly define what symptoms were considered as the first and use more standardized data as proposed by RECODE-DCM in Minimum Dataset Toolkit [40]. Moreover, identification of other significant predictors of surgical outcomes in DCM, among factors such as radiological parameters [23] or fluid biomarkers [18], may aid in surgical decision-making. In addition, factors predicting rapid deterioration are also worth further exploring to better identify patients at risk and, therefore, needing earlier surgery. The reason why some DCM patients rapidly decline, while others present long-term stability of symptoms, remains unknown.

## Conclusion

Symptom duration was demonstrated as an independent predictor of surgical outcome measured by the mJOA score. Therefore, it should be regarded as a clinically meaningful factor when counseling patients and planning surgical intervention. An optimal threshold for symptom duration, indicating when the surgery should be performed to prevent poor outcomes, cannot be recommended based on the current evidence and needs to be established by further, more standardized prospective studies. Moreover, since the natural history of disease may impact the association between symptom duration and outcome, future research is warranted to define optimal time cut-offs for different degrees of DCM severity and diverse disease trajectories.

## Supplementary Information

Below is the link to the electronic supplementary material.

Supplementary Material 1

## Data Availability

No datasets were generated or analysed during the current study.
